# A Discourse Commemorative of the Life of Prof. T. D. Mütter, M.D., LL.D.

**Published:** 1860-01

**Authors:** 

**Affiliations:** Philadelphia


A DISCOURSE COMMEMORATIVE OF THE LIFE OF PROFESSOR T. D. MUTTER, M.D., LL.D. By PROFESSOR PAN-

COAST, M.D. Philadelphia, 1859.—The discourse of Professor Pancoast will be read with great pleasure by the numerous friends and admirers of his late colleague, Dr. Mütter, who was so long one of the ornaments of the Jefferson Medical College and of the American medical profession. It was delivered as an introductory lecture on the fourteenth of October, and was published at the request of the class, one of the largest ever assembled within the walls of any medical institution. The excellent sketch of the life of Dr. Mütter, in the May number of the Review, prepared by one of his old pupils, precludes the necessity of any particular notice of Professor Pancoast's discourse.

Dr. Mütter died before he had attained the age of fifty; excessive labor, and a naturally delicate constitution, had made him prematurely old. His descent, by the father's side, was German, by the mother's, Scotch. He was early left an orphan; and, after having been a pupil at Hampden Sydney College in Virginia, he studied medicine with Dr. Simms, of Alexandria, graduating at the University of Pennsylvania in 1831. His subsequent career as a brilliant lecturer and operative surgeon is too well known to require any comments. He has left no substantial record of his experience; and his fame will be best perpetuated by his munificent bequest to the College of Physicians of Philadelphia, consisting of his museum, and of $30,000 in money for the endowment of a "surgical lectureship."

We close this brief notice with the following extracts from Professor Pancoast's discourse:—

"The first impression made by his presence was, throughout his career, always favorable. His manners were attractive, without being obsequious or abrupt, and the qualities of his heart were such as to secure him many lasting friendships. As a lecturer, his voice was clear and sonorous, and his gesticulation expressive and graceful. His fancy was full and free, and, in its brilliant play, was sometimes hard to govern. He had a desire for the possession of personal influence and position, which less ardent and more philosophic minds might deem excessive. These advantages were not coveted by him, however, for personal aggrandizement solely, but rather that he might be able to promote the interests of those who were dependent upon him or courted his support.

"The love of approbation—an aliment which seemed necessary to his sensitive nature—fed largely from his surgical successes, attached him more fondly to his avocation; made it almost the last object of his thoughts, and caused him, for the benefit of the profession and the perpetuation of his memory, to present to the College of Physicians of this city his valuable private surgical cabinet, to serve as the basis of a museum, to be called 'The Mütter Museum, founded by Thomas Dent Mütter, A.D. 1858.' A portion of his property, amounting to thirty thousand dollars, has been placed in the hands of trustees for its maintenance, and for the endowment of a lectureship on surgical pathology, and the payment of a curator. Thus, in dying, has he left a precious heritage to the profession.

"For myself especially, who lived so long in his gentle intimacy, his professional merits, great as they were, are not those which swim highest on the sea of thought. It is rather the sweetness of his character which I love most to recall; the kindness of his heart, which seldom allowed, even toward his enemies, an act of just retaliation to escape him, and I believe his colleagues, in musing over his name, will have their feelings mellowed by a similar sort of retrospection.

"Such, gentlemen, was the surgeon whom the science has lost. Such the professor, full of kindness and knowledge, whom his classes have mourned. Such the friend and colleague, torn from us in the meridian of his existence, whose memory and name we shall ever cherish."

